# A synthetic ancestral kinesin-13 depolymerizes microtubules faster than any natural depolymerizing kinesin

**DOI:** 10.1098/rsob.220133

**Published:** 2022-08-31

**Authors:** Hannah R. Belsham, Hanan M. Alghamdi, Nikita Dave, Alexandra J. Rathbone, Bill Wickstead, Claire T. Friel

**Affiliations:** ^1^ School of Life Sciences, University of Nottingham, Nottingham NG7 2UH, UK; ^2^ Biology Department, Faculty of Science, Princess Nourah bint Abdulrahman University, Riyadh, Saudi Arabia

**Keywords:** microtubule depolymerization, kinesin-13, evolution, MCAK, KIF2, ATP turnover

## Abstract

The activity of a kinesin is largely determined by the approximately 350 residue motor domain, and this region alone is sufficient to classify a kinesin as a member of a particular family. The kinesin-13 family are a group of microtubule depolymerizing kinesins and are vital regulators of microtubule length. Kinesin-13s are critical to spindle assembly and chromosome segregation in both mitotic and meiotic cell division and play crucial roles in cilium length control and neuronal development. To better understand the evolution of microtubule depolymerization activity, we created a synthetic ancestral kinesin-13 motor domain. This phylogenetically inferred ancestral motor domain is the sequence predicted to have existed in the common ancestor of the kinesin-13 family. Here we show that the ancestral kinesin-13 motor depolymerizes stabilized microtubules faster than any previously tested depolymerase. This potent activity is more than an order of magnitude faster than the most highly studied kinesin-13, MCAK and allows the ancestral kinesin-13 to depolymerize doubly stabilized microtubules and cause internal breaks within microtubules. These data suggest that the ancestor of the kinesin-13 family was a ‘super depolymerizer’ and that members of the kinesin-13 family have evolved away from this extreme depolymerizing activity to provide more controlled microtubule depolymerization activity in extant cells.

## Graphical abstract

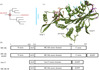

## 1. Introduction

The kinesin superfamily is a group of molecular motors that interact with the microtubule cytoskeleton. Kinesins are found in all eukaryotes, with 45 kinesin genes present in the human genome [1]. The characteristic approximately 350-residue motor domain marks a protein as a member of the kinesin superfamily. The kinesin motor domain is capable of a range of activities with respect to microtubules which, combined with the diversity of non-motor regions found within the superfamily, allow kinesins to carry out a wide array of cellular functions. Kinesin–microtubule systems underpin many vital cellular processes such as cell division, cargo transport, development and maintenance of cell polarity, growth of neuronal processes and growth and function of cilia [2–8].

Phylogenetic analysis of the kinesin superfamily has identified 17 individual families, many of which can be further divided into subfamilies [9,10]. The activity of each kinesin family is largely determined by the motor domain, and this region alone is sufficient to classify most kinesin proteins to a family. However, how the structurally highly conserved motor domain can accommodate different activities—ranging from highly processive directed movement on the microtubule [11] to non-directional short-lived diffusive motion [12], and from promoting microtubule depolymerization [13,14] to enhancing microtubule growth [15]—is not well understood. Mutational studies on individual kinesins from different families provide some insight into the residues and regions of structure in the motor domain that tailor a specific kinesin to particular activities [16–19]. While such studies have provided valuable knowledge, the extent to which they are generalizable to other motors in a subgroup or representative of the behaviour of a whole family is often unclear. This issue is exacerbated as data are often generated from a limited set of species, making the general applicability of findings to the activity of other motor domains, or to motors in phylogenetically less related organisms, difficult to ascertain. As a comprehensive approach to understanding how the motor domain sequence specifies the molecular activity of a kinesin family, we designed two reference sequences—the ‘consensus’ and the ‘ancestral’ sequences—for the motor domain of a kinesin family (kinesin-13) using a set of phylogenetically diverse eukaryotes. The ‘consensus’ motor is a sequence that exists nowhere in nature but represents a hypothetical average molecule containing the most common residues at each position across the kinesin family. The ‘ancestral’ motor is the sequence inferred as most likely to have existed in the last common ancestor of all organisms that now possess sequences belonging to the specific kinesin family.

The kinesin-13 family are a set of specialist microtubule depolymerases, with a centrally located motor domain flanked by divergent N- and C-terminal regions [20]. Kinesin-13s are major regulators of microtubule length and are particularly important during cell division [21–23]. The family is divided into three ancient subfamilies (denoted A, B and C). Subfamily B contains the most highly studied members of the family, including the mammalian kinesin-13s KIF2A, KIF2B and MCAK/KIF2C, although it is restricted to animals and their close relatives [10]. By contrast, kinesin-13A (including human KIF24) dates back to the last common eukaryotic ancestor and is the most widely distributed family. Kinesin-13s are thought to drive depolymerization by stabilizing a curved conformation of the microtubule subunit, the *α*/β-tubulin heterodimer, resulting in destabilization of the microtubule. Structures of the motor domain of either KIF2A or MCAK in complex with the *α*/β-tubulin heterodimer show that they promote curvature of tubulin larger than that observed for similar complexes of tubulin with a kinesin-1 motor domain [24–26]. The most highly studied kinesin-13, MCAK, has no translocating activity, but diffuses along the microtubule without directional bias [12]. The ability of MCAK to stabilize a curved conformation of tubulin is targeted to the microtubule end due to an atypical ATPase cycle in which only microtubule ends maximally accelerate ATP turnover [27].

By creation of reference motor domain sequences, we sought to capture the activity associated with the kinesin-13 family as a whole. The conceptual differences between the reference motors are non-trivial: the ancestral motor must have been functional but was not necessarily tuned to the specialized roles seen in extant cells. By contrast, the consensus motor could be either an optimal sequence for a family or a non-functional average. We expressed both the consensus and ancestral kinesin-13 reference motor domains and characterized their behaviour with respect to microtubules. Here we show that, while the consensus sequence acts as a microtubule depolymerase, its activity is reduced relative to MCAK. By contrast, the ancestral kinesin-13 motor domain is the fastest microtubule depolymerase studied to date, with a depolymerization rate more than an order of magnitude faster than MCAK.

## 2. Results

### 2.1. An ancestral kinesin-13 is the fastest microtubule depolymerase observed to date

**Figure 1.**
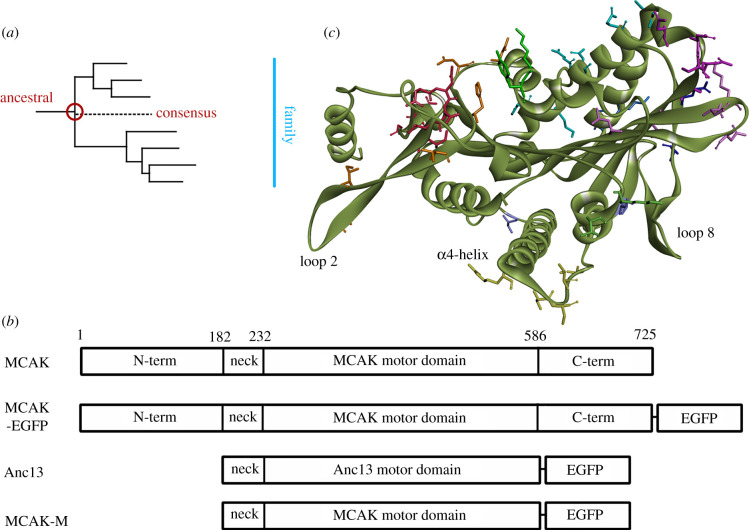
Creation of a kinesin-13 family ancestral motor domain. (*a*) Location of the ancestral-13 (Anc13) motor domain sequence on the kinesin-13 family phylogenetic tree. (*b*) Ribbon diagrams of the expression constructs used in this work: full-length MCAK (MCAK), full-length MCAK with C terminal EGFP tag (MCAK-EGFP), the ancestral-13 construct (Anc13) the human MCAK neck region is included at the N-terminal end of the Anc13 motor domain, a version of the Anc13 construct containing the MCAK motor domain in place of the Anc13 motor domain (MCAK-M). (*c*) Prediction of the structure of the Anc13 motor domain with the sidechains of residues that differ from the sequence of human MCAK shown in stick form.

To comprehensively sample the sequence space occupied by the motor domain of a kinesin family, we generated reference sequences for the motor domain of the microtubule depolymerizing kinesin-13 family from an alignment of the motor domains present in a diverse set of eukaryotes [10]. This motor domain set encompasses the widely distributed 13A subfamily (including human KIF24 and kinesin-13 sequences from plants, fungi and diverse protozoa) and the 13B subfamily, which is restricted to animals and close relatives (and includes the well-studied MCAK/KIF2C in addition to KIF2A and KIF2B). We created two representative motor domain sequences: (i) the ‘consensus’ sequence (Con13), containing the most common residue found at every sequence position in the kinesin-13 family and (ii) the ‘ancestral’ sequence (Anc13), which is the sequence from which all extant kinesin-13 proteins are inferred to have descended (i.e. the last common ancestor for the family; figure 1*a*). This latter ‘ancestral’ sequence was a maximum-likelihood estimate of the sequence existing at the root of the kinesin-13A and -13B subfamilies based on our previous phylogeny of kinesins [10] and the sequences of extant kinesins (i.e. by inferring the most likely set of ancestral states at internal nodes in the tree of kinesin-13s from diverse eukaryotic species; see Methods).

We expressed and purified protein constructs containing these motor domain sequences with the addition of the neck region of the human kinesin-13, MCAK and EGFP for visualization (figure 1*b*). The MCAK neck is included as it is shown to be required for maximal depolymerization activity of the MCAK motor domain [28,29]. Neither construct contains the N- or C-terminal regions of full-length MCAK or other kinesin-13. Dimerization of kinesin-13s is mediated by the N- and C-terminal regions. Therefore, unlike previously studied kinesin-13s, both reference constructs should be monomeric. Monomeric versions of MCAK have been shown to depolymerize microtubules but are less efficient than the full-length dimeric protein. The reference sequences share 81% identity with each other and approximately 75% identity with human MCAK across the motor domain, but do not match the sequence of any identified extant kinesin-13 (electronic supplementary material, figure S1). The differences between these reference motor domains and MCAK are not clustered, but spread throughout the motor domain, with sequence changes found in all major elements of secondary structure (figure 1*c*).

**Figure 2.**
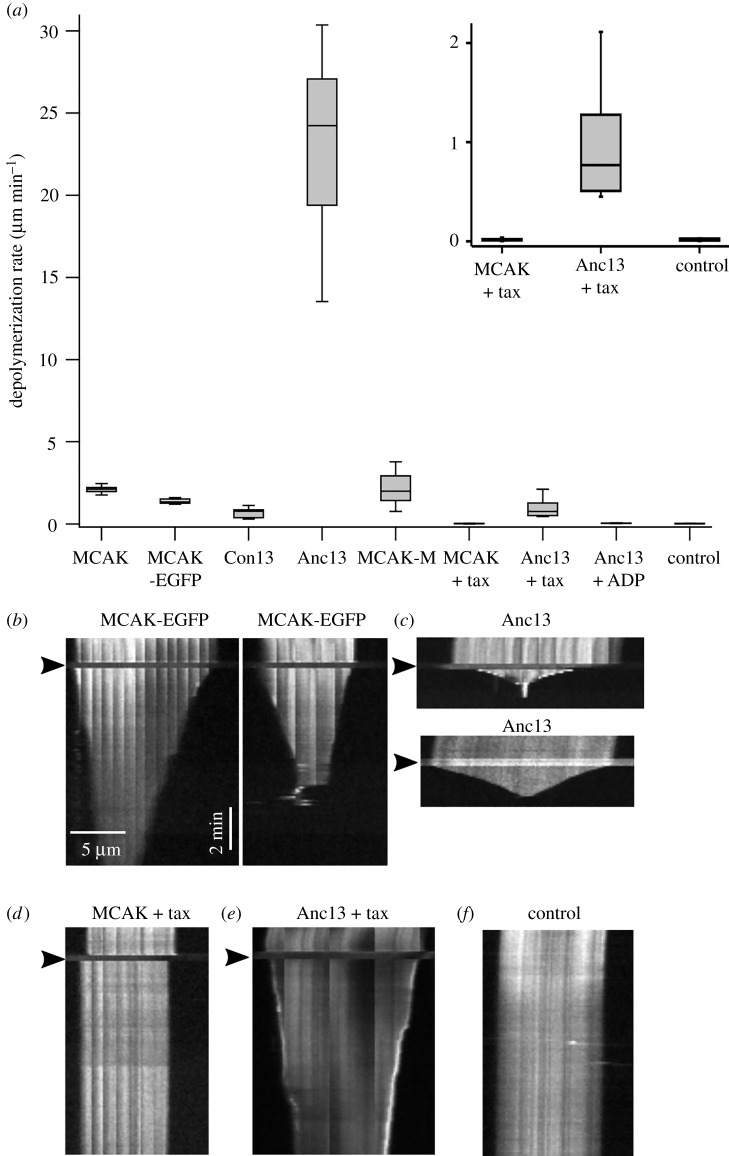
Ancestral-13 has extremely potent microtubule depolymerizing activity. (*a*) Microtubule depolymerization rates for human MCAK and EGFP compared with Con13, Anc13, MCAK-M, EGFP, Anc13 in the presence of 1 mM taxol, Anc13 in the presence of 1 mM ADP rather than ATP and a no-added-protein control. *Inset:* Zoom in on plots for MCAK and Anc13 in the presence of taxol compared with the basal microtubule depolymerization rate. (*b–f*) Representative kymographs showing the activity on individual microtubules of (*b*) EGFP, (*c*) Anc13, (*d*) EGFP + 1 mM taxol, (*e*) Anc13 + 1 mM taxol and (*f*) a microtubule with no added protein (control) showing the basal rate of depolymerization of GMPCPP-stabilized microtubules. The arrowheads indicate the time at which the specified protein was added.

We measured the depolymerization activity of these kinesin-13 reference sequences, using GMPCPP-stabilized fluorescently labelled microtubules (figure 2). Both the consensus and ancestral construct depolymerized microtubules. The depolymerization rate of the consensus motor was 0.67 ± 0.28 µm min^−1^, which is approximately 33% of the rate observed for full-length human MCAK and approximately 50% of EGFP-labelled full-length MCAK (table 1). The ancestral motor depolymerized microtubules at 23.05 ± 5.23 µm min^−1^, 11-fold faster than full-length MCAK and 17-fold faster than EGFP-labelled MCAK (figure 2*a–c*). These data make the inferred ancestral kinesin-13 motor domain the fastest microtubule depolymerase observed to date.

**Table 1.** Depolymerization rates measured for the kinesin-13 constructs used in this study given as mean ± s.d. The basal depolymerization rate of GMPCPP-stabilized microtubules in the absence of any added protein is also shown.

	microtubule depolymerization rate (µm min^−1^)
MCAK	2.12 ± 0.17 (*n* = 18)
MCAK-EGFP	1.38 ± 0.15 (*n* = 6)
Con13	0.67 ± 0.28 (*n* = 13)
Anc13	23.05 ± 5.23 (*n* = 12)
MCAK-M	2.11 ± 0.87 (*n* = 18)
MCAK + taxol	0.02 ± 0.01 (*n* = 6)
Anc13 + taxol	1.01 ± 0.59 (*n* = 12)
Anc13 + ADP	0.04 ± 0.01 (*n* = 6)
no-added-protein control (basal depolymerization)	0.02 ± 0.01 (*n* = 12)

Our investigations of Anc13 show that it requires ATP for catalytic microtubule depolymerizing activity, as is the case for all members of the kinesin-13 family studied to date [14,30]. In the presence of 1 mM ADP, Anc13 did not depolymerize microtubules (figure 2*a*). The depolymerization rate with ADP as the available nucleotide was 0.04 ± 0.01 µm min^−1^ (table 1), which is similar to the basal depolymerization rate, approximately 575-fold lower than Anc13 and over 50-fold lower than MCAK in the presence of ATP.

Full-length MCAK is a homodimer with dimerization occurring through the N- and C-terminal domains which flank the centrally located kinesin-13 motor domain [31]. The Anc13 construct does not contain the regions of full-length MCAK required for dimerization (figure 1*b*) and so is predicted to exist as a monomer. To determine if the increased depolymerization rate is due to the monomeric nature of the Anc13 motor domain rather than sequence changes within the motor domain, we created the MCAK-M construct in which the ancestral motor domain is replaced with the motor domain of human MCAK in the Anc13 construct (figure 1*b*). The microtubule depolymerization rate for this monomeric version of the MCAK motor domain is 2.11 ± 0.87 µm min^−1^ (figure 2*a* and table 1), not significantly different to full-length dimeric MCAK (*p* = 0.96) and approximately 11-fold lower than Anc13. These data indicate that, rather than differences in the oligomerization state between Anc13 and full-length MCAK, the increased rate of microtubule depolymerization for Anc13 is due to sequence changes in the motor domain.

### 2.2. Ancestral-13 depolymerizes highly stable microtubules which are impervious to MCAK

Full-length MCAK can depolymerize microtubules stabilized either by incorporation of the slowly hydrolysable GTP analogue GMPCPP or by binding of the drug Taxol, but does not depolymerize microtubules stabilized by both GMPCPP and Taxol (figure 2*a* and *d*) [27]. Double-stabilized microtubules in the presence of MCAK depolymerize at 0.02 ± 0.01 µm min^−1^ (table 1), not significantly different to the basal rate of depolymerization in the absence of added protein (figure 2*a*,*d,f*). Anc13, however, depolymerized double-stabilized microtubules at 1.01 ± 0.59 µm min^−1^ (figure 2*a,e*), 50-fold faster than the basal depolymerization rate and approximately 48% of the depolymerization rate of single-stabilized microtubules by full-length MCAK. These data show that the Anc13 motor is a more powerful depolymerase than MCAK, causing significant depolymerzation of microtubules stabilized by conditions which MCAK cannot overcome.

### 2.3. The ancestral kinesin-13 has increased microtubule affinity, but does not specifically distinguish the microtubule end

**Figure 3.**
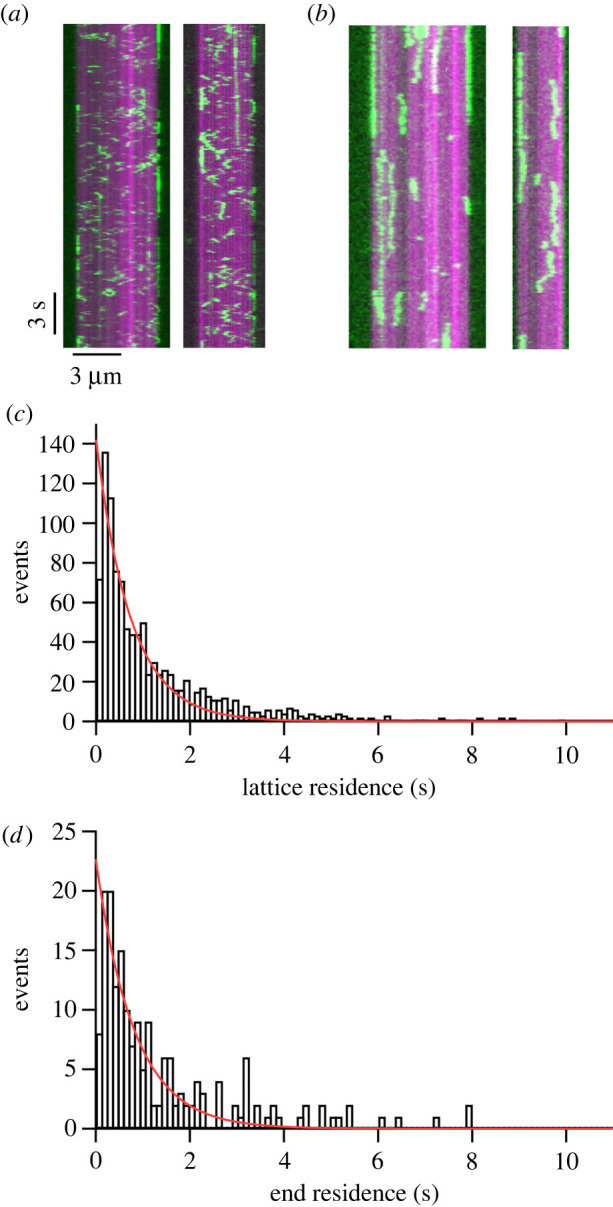
Ancestral 13 does not discriminate the microtubule end from the lattice. (*a*,*b*) Kymographs showing the interaction of (*a*) MCAK-EGFP and (*b*) Anc13 with GMPCPP-stabilized microtubules (magenta). (*c,d*) Number of events of a particular residence time for Anc13 on the (*c*) microtubule lattice and (*d*) microtubule end.

To understand how Anc13 exhibits such potent microtubule depolymerizing activity, we observed the interaction of Anc13 with microtubules using single-molecule TIRF microscopy (figure 3). The affinity for microtubules of Anc13 is much higher than MCAK. Single molecules of MCAK on microtubules are observed at low nanomolar concentrations, whereas for Anc13 we found that picomolar concentrations were required. The data shown in figure 3*a* were collected at 8 nM MCAK, whereas the data shown in figure 3*b* were collected at 40 pM Anc13. We calculated a rate of attachment to the microtubule of 62.4 ± 21.5 nM^−1^ s^−1^ µm^−1^ for Anc13, which is approximately 100-fold higher than the microtubule on-rate previously determined for MCAK (table 2) [12,16].

**Table 2.** Microtubule on and off rates for MCAK and Anc13, given as mean ± s.d., calculated using data obtained from single-molecule TIRF microscopy assays.

	*k*_on_ (um^−1^ s^−1^ nM^−1^)	*k*_off_ MT lattice (s^−1^)	*k*_off_ MT end (s^−1^)
Anc13	62.4 ± 21.5	1.36 ± 0.06	1.23 ± 0.10
MCAK	0.52 ± 0.34	2.90 ± 0.16	0.98 ± 0.06

MCAK displays mostly short (less than 1 s) diffusive interactions with the microtubule lattice. Only at microtubule ends are longer lived interactions observed. Typically, approximately 30% of MCAK end-binding events last for longer than 2 s, whereas no lattice-interaction events longer than 2 s are observed [16]. In agreement with this, the calculated *k*_off_ for MCAK when bound to the lattice is approximately threefold higher than at microtubule ends (2.90 ± 0.16 s^−1^ and 0.98 ± 0.06 s^−1^, respectively; table 2). By contrast, for Anc13 long interaction events are observed on both the microtubule lattice and end, 23% of end-binding events and 20% of lattice binding events are greater than 2 s (figure 3*b*). Although lattice-interaction events for Anc13 are longer in duration, as for MCAK, they are diffusive interactions. The off rates for the microtubule lattice and microtubule end, calculated for Anc13 are similar, *k*_off (lattice)_ = 1.36 ± 0.06 s^−1^ and *k*_off (end)_ = 1.23 ± 0.10 s^−1^. The ratio of *k*_off (lattice/end)_ for Anc13 is 1.11 ± 0.14, not significantly different from 1, indicating that, unlike MCAK, Anc13 does not discriminate between the microtubule end and the lattice.

### 2.4. Ancestral kinesin-13 has a high basal rate of ATP turnover which is relatively insensitive to the presence of microtubules

To understand how the turnover of ATP is coupled to microtubule depolymerization by Anc13, we measured ATP turnover in solution, in the presence of unpolymerized tubulin and in the presence of GMPCPP-stabilized microtubules (table 3). The solution/basal ATPase for Anc13 was 0.44 ± 0.04 s^−1^, which is approximately 200-fold higher than for full-length MCAK (table 3). This high rate of ATP turnover in the absence of tubulin could be due to the lack of the N- and C-terminal domains, which have been shown to interact with the motor domain of MCAK and may inhibit ATP turnover [32,33]. However, the basal ATPase for MCAK-M, the MCAK motor domain lacking the N- and C-terminal regions, was 0.003 ± 0.001 s^−1^, not significantly different from full-length MCAK (*p* = 0.25).

**Table 3.** ATPase rates for MCAK and Anc13, given as mean ± s.d., measured in solution (basal rate in the absence of tubulin), in the presence of unpolymerized tubulin and in the presence of microtubules.

	ATPase (s^−1^)		
	in solution	+unpolymerized tubulin	+microtubules
Anc13	0.44 ± 0.04 (*n* = 3)	3.76 ± 0.81 (*n* = 8)	8.07 ± 0.63 (*n* = 3)
MCAK	0.002 ± 0.001 (*n* = 4)	0.25 ± 0.10 (*n* = 7)	3.69 ± 0.38 (*n* = 4)

ATP turnover by Anc13 was accelerated by unpolymerized tubulin. The ATPase rate for Anc13 in.the presence of 10 µM tubulin was 3.76 ± 0.81 s^−1^, an increase of approximately eightfold over the basal ATPase. However, this fold increase in ATP turnover is small compared with MCAK for which unpolymerized tubulin causes an approximately 100-fold increase over the basal rate of ATP turnover [27].

The fold acceleration in ATP turnover caused by the presence of microtubules is also small for Anc13 compared with MCAK (table 3). While the microtubule-stimulated ATPase for MCAK is accelerated approximately 2000-fold with respect to the basal ATPase, the microtubule-stimulated ATPase for Anc13 is 8.07 ± 0.63 s^−1^, only an approximately 20-fold increase over the basal ATPase.

These data show that the ancestral kinesin-13 construct has a high rate of ATP turnover even in the absence of tubulin, whereas the MCAK motor domain placed in the same context, as part of the MCAK-M construct, has a very low basal ATPase similar to full-length MCAK. This indicates that the high basal ATPase of Anc13 is an intrinsic property of this motor domain, rather than a result of the absence of the N- and C-terminal regions that flank the motor domain in full-length MCAK.

### 2.5. The ancestral-13 motor domain, placed within a full-length kinesin-13, retains potent depolymerization activity and promotes internal breaking of microtubules

**Figure 4.**
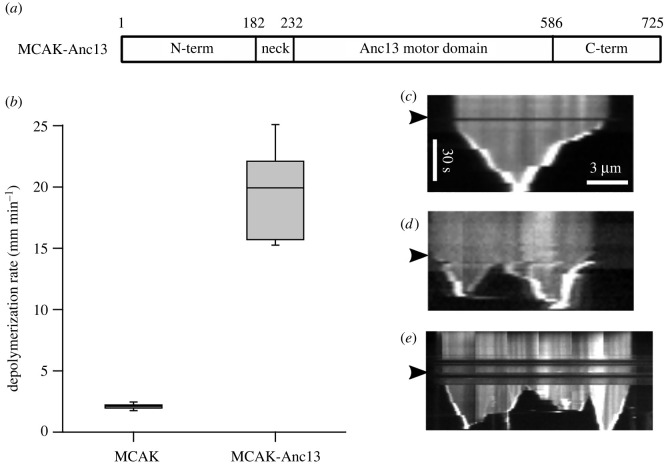
The ancestral-13 motor domain in the context of full-length MCAK has extremely potent microtubule depolymerizing activity. (*a*) Ribbon diagram of the expression construct in which the ancestral-13 motor domain is placed within full-length MCAK. Residues 1–232 and 586–725 are the sequence of WT-MCAK. (*b*) Microtubule depolymerization rates for MCAK and MCAK-Anc13. (*c–e*) Representative kymographs showing the activity on individual microtubules of MCAK-Anc13. (*d,e*) Kymographs showing examples of microtubule which break and continue to depolymerize from the new internally generated ends. The arrowheads indicate the time at which MCAK-Anc13 was added.

To understand the behaviour of the ancestral-13 motor domain within the context of a full-length extant kinesin-13, we created a construct in which the motor domain of full-length wild-type MCAK was replaced with the ancestral-13 motor domain, MCAK-Anc13 (figure 4*a*). This construct depolymerized GMPCPP-stabilized microtubules at 19.50 ± 4.87 µm min^−1^, approximately ninefold faster than the depolymerization rate for MCAK and not significantly different (*p* = 0.1) from the depolymerization rate observed for the Anc13 construct (figure 4*b,c*). These data indicate that the ancestral-13 motor retains ultra-rapid depolymerization activity when placed in the context of full-length MCAK, the addition of surrounding N- and C-terminal regions does not significantly alter its microtubule depolymerization activity.

In contrast with both MCAK and Anc13, the MCAK-Anc13 construct was observed to promote breaking of GMPCPP-stabilized microtubules leading to depolymerization from internal sites (figure 4*d,e*). This activity is not frequently observed when microtubules are subjected to depolymerization by MCAK, which typically causes depolymerization only from microtubule ends. Quantification of the frequency of internal breaking for microtubules longer than 5 μm showed that in the presence of MCAK approximately 5% of microtubules (*n* = 58) broke at internal sites, while in the presence of MCAK-Anc13 approximately 70% of microtubules (*n* = 47) broke and began to depolymerize from these breaks at a similar rate as from the original microtubule ends. These data indicate that the ancestral motor is an extremely potent depolymerase both as a synthetic monomeric construct and as a dimer in the context of a full-length kinesin-13.

## 3. Discussion

### 3.1. The ancestral kinesin-13 motor domain is a powerful, ultra-rapid microtubule depolymerase

The design and study of reference sequences for the kinesin-13 motor domain provide insight into the evolution of the kinesin-13 family and generalized information on the activity of the family as a whole. Both the consensus and ancestral motor domains have microtubule depolymerizing activity and depolymerize microtubules from both ends. The consensus sequence (Con13), which contains the most common residue at each position across the family, depolymerizes microtubules at approximately 1/3 the rate of our comparator MCAK. This agrees with the assumption that all members of the kinesin-13 family are microtubule depolymerases.

In contrast with the consensus protein, the microtubule depolymerization rate for the ancestral kinesin-13 motor domain (Anc13) is an order of magnitude faster than the most highly studied member of the kinesin-13 family, MCAK. To our knowledge, this synthetic protein is the fastest microtubule depolymerase observed to date. In fact, Anc13 has such potent activity that it can depolymerize microtubules under stabilizing conditions in which MCAK has no effect. These observations suggest that the founding members of the kinesin-13 family had much more potent depolymerase activity than kinesin-13s currently existing in nature (or at least those studied to date). Of course, this activity may have been tempered in ancestral cells by tight regulation of expression or localization, or by inhibition by regions adjacent to the motor domain that can no longer be reconstructed by phylogenetic analysis. However, potent depolymerase activity is retained when the ancestral-13 motor domain is placed in the context of full-length MCAK surrounded by the non-motor N- and C-terminal regions. It appears that kinesin-13s in extant lineages have not evolved to become ‘better’ depolymerizers but have instead evolved away from ultra-rapid ‘super’ depolymerase activity towards milder action.

### 3.2. Ultra-rapid depolymerization is associated with inability to focus activity to microtubule ends

Single-molecule TIRF data show that, while Anc13 has higher overall affinity for microtubules, it does not distinguish between the microtubule end and the microtubule lattice. Rather, Anc13 displays long binding events at all positions on the microtubule. This contrasts with MCAK for which long binding events are observed only at or near microtubule ends [16], focusing activity to the place in which tubulin removal can most easily occur and protecting the microtubule body from destabilization. The microtubules used here are GMPCPP-stabilized and so the microtubule ends are not chemically distinct from the lattice as regards bound nucleotide. This implies that the ability of MCAK to distinguish the microtubule end from the lattice is based upon detection of differences in the configuration of tubulin, such as bending or increased conformational freedom near the microtubule end. The ultimate extension of this lack of ability to focus activity to the microtubule end is observed for the MCAK-Anc13 construct, which causes internal breaks within microtubules and promotes depolymerization not only from the microtubule end, but also from these break sites. This type of behaviour is rarely observed for extant kinesin-13s but resembles the activity of known microtubule severing proteins such as katanin, although the molecular mechanism is likely different [34]. MCAK-Anc13 appears to recognize and be able to promote depolymerization from sites of minimal configurational difference that are not recognized by MCAK. However, the internal microtubule breaks promoted by MCAK-Anc13 may occur at existing defects within the microtubule.

### 3.3. Ancestral kinesin-13 removes multiple tubulin dimers per ATP consumed

The stoichiometry of ATP consumed per tubulin removed by MCAK has previously been measured to range from 1 ATP to greater than 20 ATPs per tubulin [27]. During processive depolymerization of GMPCPP-stabilized microtubules MCAK removes on average 1 tubulin for each ATP consumed: every ATPase cycle is productive for depolymerization. With progressively more stable microtubules MCAK fails to remove tubulin for some ATP turnover cycles. Thus the proportion of productive cycles decreases from 100% for GMPCPP-stabilized microtubules, to 50% for taxol stabilized microtubules and to less than 5% for double-stabilized microtubules [27,30]. However, from currently available data, there is no indication that MCAK removes more than 1 tubulin dimer on average per ATP under any conditions.

The observation that the microtubule-stimulated ATPase for Anc13 was only approximately twofold higher than MCAK while the microtubule depolymerization rate was greater than 10-fold higher was unexpected, as this indicates that Anc13 removes more than a single tubulin for each cycle of ATP turnover. The stoichiometry of ATP consumed per tubulin removed for Anc13 is approximately 0.2 ATP per tubulin, indicating that for each ATP consumed Anc13 removes multiple tubulin dimers. From our data, we calculate that Anc13 removes on average 5.8 ± 1.4 tubulins per ATP, when acting on GMPCPP-stabilized microtubules.

**Figure 5.**
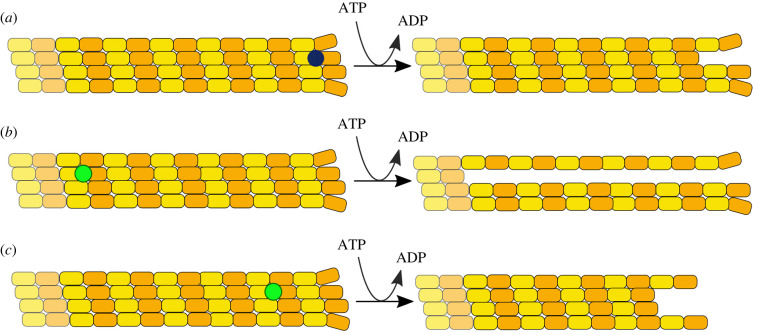
Microtubule depolymerization by MCAK compared with possible mechanisms of depolymerization by Anc13. Schematic of a microtubule depolymerized by (*a*) MCAK and (*b,c*) Anc13. (*a*) Available data indicate that MCAK typically binds to and removes only the terminal tubulin dimer. The highest ratio of tubulin removed to ATP consumed measured for MCAK is 1 : 1. MCAK on average removes a single tubulin for each ATP consumed. (*b*,*c*) Our data show that Anc13 removes on average 5.8 ± 1.4 tubulin dimers per ATP consumed. This suggests that Anc13 binds to and destabilizes tubulin dimers prior to the terminal protofilament dimer. (*b*) Anc13 could bind to the sixth dimer prior to a protofilament end and cause detachment of the rest of the same protofilament, or (*c*) Anc13 could bind closer to the protofilament end and cause detachment of tubulin subunits from the same protofilament plus tubulin from neighbouring protofilaments.

To explain these data, Anc13 must be able to bind at locations prior to the terminal tubulin dimer and destabilize tubulin subunits relatively distant to the microtubule end (figure 5). Our data indicate that on average six tubulin dimers are removed per single cycle of ATP turnover. This could mean that Anc13 on average binds six dimers away from the microtubule end and causes detachment of the bound tubulin and the remaining subunits from the same protofilament (figure 5*b*) or, perhaps more likely, that Anc13 binds anywhere up to six dimers away from the microtubule end and causes destabilization of the bound tubulin leading to detachment of the remaining subunits from the same protofilament plus destabilization of tubulin subunits from neighbouring protofilaments, causing on average six dimers to detach from the microtubule (figure 5*c*).

Tubulin dimers farther from the microtubule end are probably more stably embedded within the microtubule than those close to the end. Therefore, to destabilize tubulin subunits at sites prior to the terminal dimer of a microtubule protofilament would require higher affinity binding to deform and destabilize tubulin dimers located at more stable positions within the microtubule. Anc13 not only binds microtubules with higher affinity than MCAK, but also can depolymerize microtubules under stabilizing conditions that cannot be overcome by MCAK. Further, when the ancestral-13 motor domain acts as a dimer in the context of full-length MCAK, it causes breaking of microtubules at internal positions. These data indicate that the ancestral-13 motor domain can promote deformation and destabilization of tubulin subunits distant to the microtubule end resulting in the removal of multiple dimers per ATP turnover cycle and internal breaking of microtubules, resulting in the most rapid microtubule depolymerase activity observed to date.

### 3.4. The kinesin-13 family evolved away from potent activity towards more controlled microtubule depolymerization

Our data suggest that the founding members of the kinesin-13 family were highly potent, ‘super’ depolymerases. The comparison of the activity of a phylogenetically inferred ancestral kinesin-13 motor domain with extant kinesin-13s implies that as the family developed its members evolved away from rampant depolymerization towards more controlled, but less rapid, activity. The comparison of the biochemical parameters for Anc13 with those previously determined for MCAK shows that while Anc13 can depolymerize microtubules an order of magnitude faster, this comes at the cost of efficiency in terms of ATP turnover and specificity in localization.

It is not obvious which sequence changes between Anc13 and MCAK are responsible for the increased potency of the ancestral-13 motor domain. Sequence changes are scattered throughout the motor domain (electronic supplementary material, figures S1 and S2) and no sequence alterations are found in any of the conserved kinesin-specific nucleotide-binding motifs or in the kinesin-13-family-specific microtubule-binding motifs.

In comparison to the ancestral sequence, modern-day MCAK has gained several characteristics that are presumably beneficial for the physiological roles of extant members of the kinesin-13 family. Mitotic spindle and flagellar length control are proposed to be the principal roles of kinesin-13s conserved throughout the evolution of eukaryotes [35]. Highly potent, ultra-rapid depolymerization that is not focused at microtubule ends but causes internal breaking of microtubules, is likely not beneficial for these roles which require precise regulation of microtubule length. Our data suggest that better-regulated activity has outcompeted rapid but less focused depolymerization activity during the evolution of the kinesin-13 family. The design and creation of bioinformatically inferred reference sequences provide insight into the evolution of the kinesin-13 family and could provide similar insight into other kinesin families or other protein families. The approach described allows comprehensive sampling of sequence space, which could provide insight into the evolution of function for any large paralogous protein family.

## 4. Methods

### 4.1. Inference of reference motor domain sequences

Reference sequences for the kinesin-13 family were based on the previous analysis of 1624 kinesins proteins encoded in the genomes of 45 diverse extant eukaryotes [10]. The alignment of the motor domains and the phylogenetic tree for the kinesin-13 family were extracted from this larger analysis of the full kinesin superfamily. The kinesin-13 subtree encompasses 92 protein sequences from 38 eukaryotes (i.e. all those in the analysis that encode a kinesin-13 family member). To avoid reconstruction being heavily biased by divergent sequences, the 13C subfamily (which includes divergent kinesin-13-like sequences from various microbial eukaryotes) was excluded and only subfamilies 13A and 13B were considered (75 sequences in total) in both consensus and ancestral sequence inference.

Consensus motor domain sequence was inferred by a simple most common residue method, with ties resolved based on most likely residue by BLOSUM62 score. Ancestral sequences were inferred by maximum likelihood from the kinesin-13A/B subtree and alignment of extant motor domain sequences using the WAG substitution matrix as implemented by fastML v. 3.1 (–seqType aa –SubMatrix WAG –indelReconstruction ML) [36]. A single internal polytomy in the 13B subfamily tree was resolved based on branching of the included species, although this has no impact on inference of the ancestral state of the 13A/B family. The tree used for ancestral sequence reconstruction and full alignment of motors for the kinesin-13A/B subfamilies are provided in the electronic supplementary material, figure S3 and file S1, respectively.

### 4.2. Expression and purification of kinesin reference constructs

Reference protein sequences were reverse translated using a *Spodoptera frugiperda* (Sf9) codon usage table (IDT Codon Optimization Tool, www.idtdna.com). These coding sequences were incorporated into derivatives of pFastBac1 encoding the neck region from Hs MCAK on the N-terminal side and eGFP and TEV-cleavable streptavidin-binding peptide- and His-tags on the C-terminal side (see electronic supplementary material, files S2 and S3 for full plasmid sequences). The formation of the expected chimeric sequence was confirmed by Sanger sequencing covering the full CDS.

The proteins were expressed in Sf9 cells (Bac-To-Bac expression system; Invitrogen). Cells were lysed by incubation at 4°C for 1 h in 50 mM Tris pH7.5, 300 mM NaCl, 10% glycerol, 5 mM MgCl_2,_ 0.1% Tween 20, 10 mM imidazole, 1 mM DTT and protease inhibitors. The proteins were purified by Ni-affinity chromatography (Ni-NTA agarose, Qiagen) followed by StrepTactin affinity chromatography (StrepTactin Sepharose high performance, GE Healthcare).

### 4.3. Microtubules and depolymerization assays

Porcine brain tubulin was purified from homogenized brain tissue using the high ionic strength method [37]. Microtubule depolymerization rates were determined by measuring the length of immobilized, GMPCPP-stabilized, 25% rhodamine-labelled microtubules over time after the addition of 40 nM kinesin-13 as described previously [16]. These buffer conditions and concentration have previously been shown to be optimal for MCAK activity. The depolymerization data for MCAK-Anc13 was captured by imaging every 2 s, rather than every 5 s, as was the case for all other proteins studied.

### 4.4. Single-molecule TIRF microscopy

Rhodamine-labelled, GMPCPP-stabilized microtubules were adhered to the surface of flow chambers prepared as described previously [38]. MCAK-eGFP or Anc13 at single-molecule concentrations (4–8 nM and 40 pM, respectively) were added to a microtubule containing flow cell in BRB20 pH 6.9, 75 mM KCl, 1 mM ATP, 0.05% Tween 20, 0.1 mg ml^−1^ BSA, 1% 2-mercaptoethanol, 40 mM D-glucose, 40 mg ml^−1^ glucose oxidase and 16 mg ml^−1^ catalase. Images were recorded using a Zeiss PS1 Super Resolution microscope. Microtubules were imaged using laser excitation (561 nm) and a 579–620 nm emission filter. Movies of eGFP-labelled single molecules were collected using TIRF excitation at 488 nm and a 495–550 emission filter, 200 images were collected per movie with a frame rate of 8.7 Hz.

For analysis, each frame of the GFP dataset was colour-combined with the corresponding image of rhodamine-labelled microtubules in FIJI to enable identification of on-microtubule events. Kymographs for individual microtubules were used to measure the duration of individual GFP localization events at the microtubule end and on the lattice, also in FIJI [39].

### 4.5. ATPase assays

ATPase rates in solution were measured using 3 µM kinesin by monitoring the production of ADP using HPLC as described previously [40]. For assays with added tubulin or microtubules, 0.1 µM kinesin was used and the production of ADP monitored by linking it to the oxidation of NADH [16,41]. For both assays, the change in concentration of ADP per second was divided by the concentration of kinesin motor domain to give the ATPase activity per second per active site.

## Data Availability

Data are presented as figures within the manuscript. Raw data not included within the manuscript are available as electronic supplementary material [42] or from the authors.
